# Molecular signature of anastasis for reversal of apoptosis

**DOI:** 10.12688/f1000research.10568.2

**Published:** 2017-02-09

**Authors:** Ho Man Tang, C. Conover Talbot Jr, Ming Chiu Fung, Ho Lam Tang

**Affiliations:** 1 Institute for Basic Biomedical Sciences, Johns Hopkins University School of Medicine, Baltimore, USA; 2 School of Life Sciences, Chinese University of Hong Kong, Shatin, Hong Kong; 3 W. Harry Feinstone Department of Molecular Microbiology and Immunology, Johns Hopkins University Bloomberg School of Public Health, Baltimore, USA

**Keywords:** Anastasis, apoptosis, Cell Death, Cell Survival, Gene Expression, Recovery, Repair, Reversal of Apoptosis

## Abstract

Anastasis (Greek for "rising to life") is a cell recovery phenomenon that rescues dying cells from the brink of cell death. We recently discovered anastasis to occur after the execution-stage of apoptosis *in vitro* and *in vivo*. Promoting anastasis could in principle preserve injured cells that are difficult to replace, such as cardiomyocytes and neurons. Conversely, arresting anastasis in dying cancer cells after cancer therapies could improve treatment efficacy. To develop new therapies that promote or inhibit anastasis, it is essential to identify the key regulators and mediators of anastasis – the therapeutic targets. Therefore, we performed time-course microarray analysis to explore the molecular mechanisms of anastasis during reversal of ethanol-induced apoptosis in mouse primary liver cells. We found striking changes in transcription of genes involved in multiple pathways, including early activation of pro-cell survival, anti-oxidation, cell cycle arrest, histone modification, DNA-damage and stress-inducible responses, and at delayed times, angiogenesis and cell migration. Validation with RT-PCR confirmed similar changes in the human liver cancer cell line, HepG2, during anastasis. Here, we present the time-course whole-genome gene expression dataset revealing gene expression profiles during the reversal of apoptosis. This dataset provides important insights into the physiological, pathological, and therapeutic implications of anastasis.

## Introduction

Apoptosis (Greek for “falling to death”) is essential for normal development and homeostasis of multicellular organisms by eliminating unwanted, injured, or dangerous cells ^1– 3^. This cell suicide process was generally assumed to be irreversible because it involves rapid and massive cell destruction ^4– 9^. During apoptosis, intrinsic and extrinsic pro-apoptotic signals can converge at mitochondria, leading to mitochondrial outer membrane permeabilization (MOMP), which releases cell execution factors, such as cytochrome *c* to trigger activation of apoptotic proteases including caspase-3 and -7 ^10, 11^, small mitochondria-derived activator of caspases (Smac)/direct IAP binding protein with low pI (DIABLO) to eliminate inhibitor of apoptosis protein (IAP) which suppresses caspase activation ^12, 13^, and apoptosis-inducing factor (AIF) and endonuclease G to destroy DNA ^14– 17^. Activated caspases commit cells to destruction by cleaving hundreds of functional and structural cellular substrates ^4, 18^. Crosstalk between signalling pathways amplifies the caspase cascade to mediate cell demolition via nucleases (DNA fragmentation factor [DFF]/caspase-activated DNase [CAD]) to further destroy the genome ^19– 21^, and alter lipid modifying enzymes to cause membrane blebbing and apoptotic body formation ^22, 23^. Therefore, cell death is considered to occur after caspase activation within a few minutes ^24– 26^.

However, we and other groups have demonstrated reversal of early stage apoptosis, such as externalization of phosphatidylserine (PS) in cultured primary cells and cancer cell lines ^27– 30^. We have further demonstrated that dying cells can reverse apoptosis even after reaching the generally assumed “point of no return” ^29– 31^, such as MOMP-mediated cytochrome *c* release, caspase-3 activation, DNA damage, nuclear fragmentation, and apoptotic body formation ^4– 9^. Our observation of apoptosis reversal at late stages is further supported by an independent study, which shows recovery of cells after MOMP ^32^. To detect reversal of apoptosis in live animals, we have further developed a new *in vivo* caspase biosensor, designated “CaspaseTracker” ^33^, to identify and track somatic, germ and stem cells that recover after transient cell death inductions, and also potentially during normal development and homeostasis in *Drosophila melanogaster* after caspase activation ^33, 34^, the hallmark of apoptosis ^4, 35^. We proposed the term “anastasis” ^30^, which means “rising to life” in Greek, to describe this recovery from the brink of cell death. Anastasis appears to be an intrinsic cell survival phenomenon, as removal of cell death stimuli is sufficient to allow dying cells to recover ^29– 31, 33^.

The physiological, pathological and therapeutic importance of anastasis is not yet known. We proposed that anastasis could be an unexpected tactic that cancer cells use to escape cancer therapy ^29– 31^. Many tumours undergo dramatic initial responses to cell death-inducing radiation or chemotherapy ^36– 39^; however, these cells relapse, and metastasis often occurs in most types of cancer ^36– 39^. Therefore, the ability of cells to recover from transient induction of cell death may allow tumour cells to escape treatment, and survive and proliferate, resulting in relapse ^29– 31^. Furthermore, cells may acquire new oncogenic mutations and transformation phenotypes during anastasis ^30, 31^, such as DNA damage caused by apoptotic nucleases. Therefore, anastasis could be one of the mechanisms underlying the observation that repeated tissue injury increases the risk of cancer in a variety of tissues ^40^, such as liver damage due to alcoholism ^41^, chronic thermal injury in the oesophagus induced by the consumption of very hot beverages ^42– 44^, evolution of drug resistance in recurrent cancers ^36– 39, 45^, and development of a second cancer during subsequent therapy ^46– 49^. Anastasis can also occur in primary cardiac cells and neuronal cell lines ^30, 31^, and potentially in cardiomyocytes *in vivo* following transient ischemia ^50^. These findings suggest anastasis as an unexpected cellular protective mechanism. Therefore, uncovering the mechanisms of anastasis may provide new insights into the regulation of cell death and survival, and harnessing this mechanism via suppression or promotion of anastasis would aid treatment of intractable diseases including cancer, heart failure and neurodegeneration.

Our previous study demonstrated reversibility of ethanol-induced apoptosis at late stages in mouse primary liver cells, and revealed that new transcription is important to reverse apoptosis ^30, 31^. During recovery, we found up-regulation of genes involved in pro-survival pathways and DNA damage responses during anastasis (Bag3, Mcl1, Dnajb1, Dnajb9, Hsp90aa1, Hspa1b, and Hspb1, Mdm2) ^30^. Interestingly, inhibiting some of those genes by corresponding specific chemical inhibitors significantly suppresses anastasis ^30^. However, the molecular mechanism of anastasis remains to be elucidated. To study the cellular processes of anastasis, we performed time-course RNA microarray analysis to determine the gene expression profiles of the cultured mouse primary liver cells undergoing anastasis following transient exposure to ethanol that triggers apoptosis, and identified unique gene expression patterns during reversal of apoptosis. We also performed reverse transcription polymerase chain reaction (RT-PCR) to validate the gene expression patterns in the human liver cancer cell line, HepG2, during anastasis. Here, we present our time-course microarray data, which reveals the molecular signature of anastasis.

## Methods

### Microarray

Mouse primary liver cells were isolated from BALB/c mice using collagenase B and cultured as described ^30, 51^. The cells were cultured in in DMEM/F-12 (DMEM:nutrient mixture F-12) supplemented with 10% fetal bovine serum (FBS), 100 U/ml penicillin, and 100 μg/ml streptomycin (Life Technologies, Carlsbad, CA, USA) at 37°C under an atmosphere of 5% CO _2_/95% air. To induce apoptosis, cells were exposed to 4.5% ethanol for 5 hours (R0) in the culture medium (vol/vol). To allow recovery, dying cells were washed and further incubated in the fresh culture medium for 3 hours (R3), 6 hours (R6), 24 hours (R24), and 48 hours (R48). The untreated cells served as control (Ctrl). Three biological replicates were performed at each time point. Total RNA in the corresponding cell conditions was harvested using TRIzol Reagent (Life Technologies). The RNA was purified using the RNeasy Mini Kit (Qiagen, Cologne, Germany). Reverse transcription was performed using SABiosciences C-03 RT ^2^ First Strand Kit to construct cDNA (SABiosciences-Qiagen, Frederick, MD, USA). The cDNA samples were analysed using the Illumina MouseWG-6 v2.0 Expression BeadChip (Illumina, San Diego, CA, USA).

### Gene expression data analysis

The Partek Genomics Suite 6.6 (Partek, St. Louis, MO, USA) was used for principal component analysis (PCA) ^52, 53^. The Spotfire DecisionSite 9.1.2 (TIBCO, Palo Alto, CA, USA) platform was used to evaluate the fold change of gene expression levels between time points when compared with a common starting point, which is the control (Ctrl) ^54^. Signal values were converted into log _2_ space and quality control tests were performed to ensure data integrity by comparing the signals of the three biological replicates at each time point. The fold change was based on averaged values of the three replicates at each time point; two-sample Student's *t*-test was used to determine statistical significance as *p*-values of less than 0.05, using the Partek Genomics Suite v6.5 (Partek Inc., St. Louis, MO, USA).

For the time-course gene expression analysis using Spotfire, all time points were compared with the time point Ctrl, which represents untreated cells. Spotfire was used to show the genes that displayed specific changes in gene expression after removal of cell death inducer for 3 hours (R3) and 6 hours (R6). Genes with specific and significant change (Log _2 _> 1 or <-1) in expression at the corresponding timepoint are highlighted. Interaction network analysis of the up-regulated genes during anastasis was performed using the GeneMANIA database ( http://genemania.org/) ^55, 56^.

### Confocal microscopy

Cells were incubated with 50 nM Mitotracker Red CMXRos and 250 ng/ml Hoechst 33342 (Invitrogen) for 20 minutes in culture medium to stain mitochondria and nuclei, respectively. The stained cells were washed and incubated with culture medium for 10 minutes, and then were fixed with 3.7% (wt/vol) paraformaldehyde in phosphate-buffer saline (PBS) solution for 20 minutes at room temperature in dark. The fixed cells were mounted on glass slide using ProLong Diamond Antifade Mountant (Invitrogen). Cell images were captured with the Zeiss LSM 780 confocal inverted microscope using a 40×, numerical aperture (NA) 1.4 plan-Apochromat objective (Carl Zeiss, Jena, Germany), and were analyzed using Zen 2013 or AxioVision 4.2 software (Carl Zeiss).

### Reverse transcription polymerase chain reaction (RT-PCR)

Human liver cancer cell line HepG2 (ATCC HB-8065) was cultured in DMEM/F-12, 10% FBS, 100 U/ml penicillin, and 100 μg/ml streptomycin (Life Technologies) at 37°C under an atmosphere of 5% CO _2_/95% air. Apoptosis was induced by incubation of the cells with 4.5% ethanol in cell culture medium for 5 hours (R0). Then, the apoptotic dying cells were washed and then incubated in the fresh culture medium for 1 hour (R1), 2 hours (R2), 3 hours (R3), 4 hours (R4), 6 hours (R6), 9 hours (R9), 12 hours (R12), and 24 hours (R24). The untreated cells served as control (Ctrl). Total RNA in the corresponding cell conditions was harvested using QIAzol lysis reagent (Qiagen). The total RNA was purified using the RNeasy Mini Kit (Qiagen). Reverse transcription was performed using the SuperScript IV reverse transcriptase system (Thermo Fisher Scientific, Waltham, MA, USA). Primer sets for detecting targeted genes were designed using the Universal ProbeLibrary (Roche Applied Science, Madison, WI). Primer set for MMP10 was previously designed ^57^. Polymerase Chain Reaction (PCR) was performed using Taq DNA Polymerase and PCR protocol (New England BioLabs, Ipswich, MA, USA), with initial denaturation at 95°C for 2 minutes, followed by 30 cycles of denaturation at 95°C for 30 seconds, annealing at 60°C for 30 seconds, and extension at 72°C for 3 seconds. Electrophoresis of PCR products was performed using 4% agarose gel.

## Results and discussion

**Figure 1.**
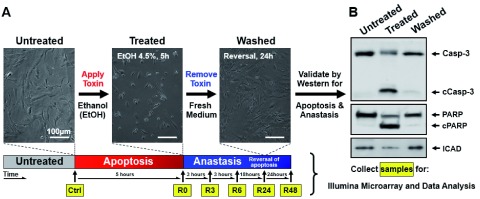
Flow chart for experimental design. Mouse primary liver cells were treated with 4.5% ethanol for 5 hours (R0) and then washed and cultured in fresh medium for 3 (R3), 6 (R6), 24 (R24), and 48 (R48) hours. The untreated cells served as control (Ctrl). ( **A**) Time-lapse live-cell light microscopy and ( **B)** Western blot analysis validated apoptosis to occur at R0, and anastasis at R24. Cells were collected at the indicated timepoints of ( **A**) for RNA extraction. Gene expression profiling was performed by microarray, and analysed by Spotfire. The images from Figure 1A and B are adopted from the *Mol Biol Cell* 23, 2240–52 (2012) ^30^. Reprinted with permission.

**Figure 2.**
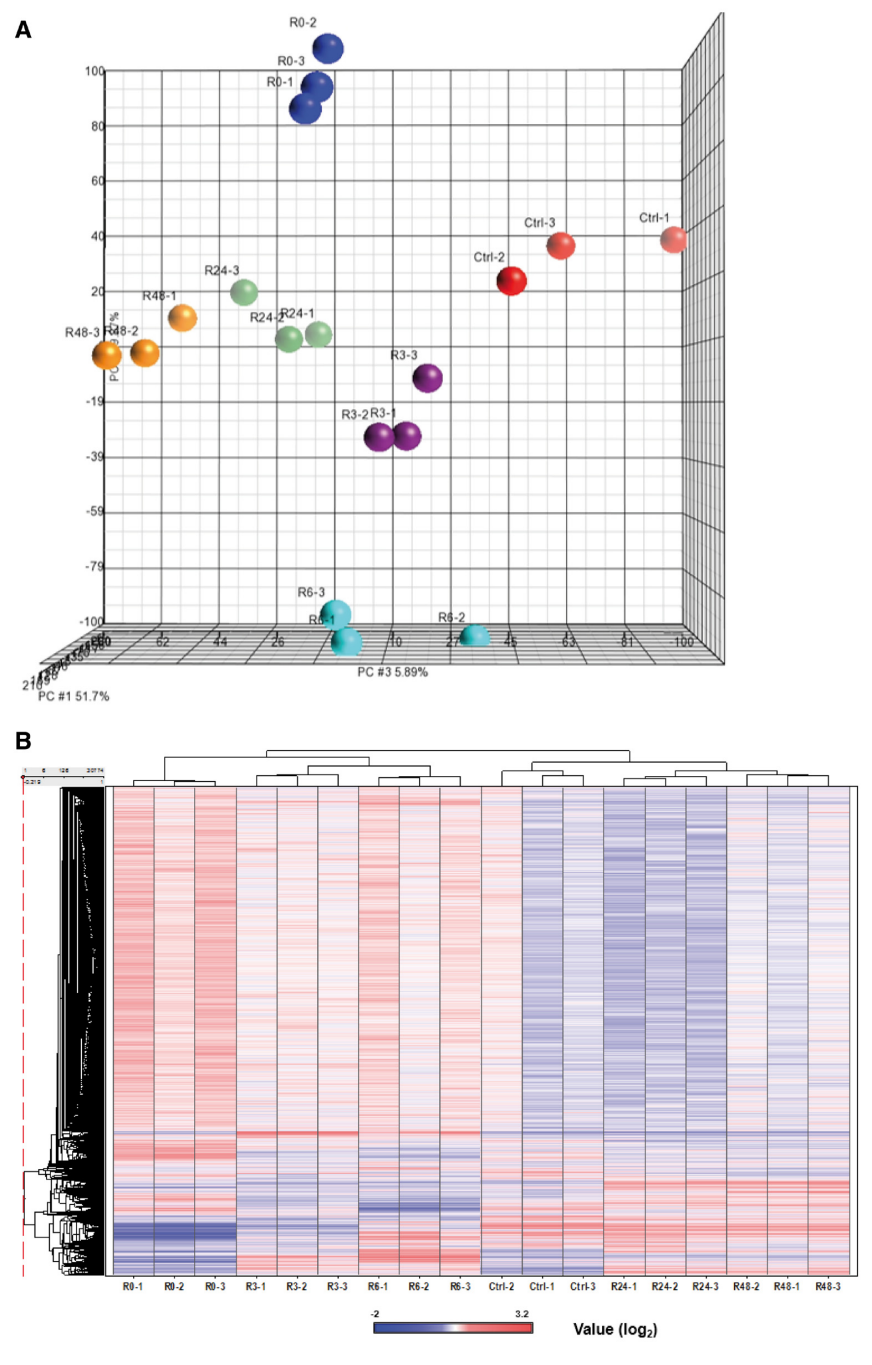
Technical validation of microarray data. The three biological replicate samples of microarray data were shown to cluster together by using ( **A**) principal component analysis (PCA) and ( **B**) unsupervised hierarchical clustering of the RNA microarray data of eighteen samples.

We have demonstrated that mouse primary liver cells can reverse the apoptotic process at the execution stage ^30, 31^, despite reaching important checkpoints commonly believed to be the “point of no return” ^4– 9^, including caspase-3 activation, DNA damage, and cell shrinkage. To pursue the mechanisms of anastasis, we performed time-course high-throughput microarray to evaluate gene expression profiles during reversal of ethanol-induced apoptosis in mouse primary liver cells. RNA samples were collected from the untreated primary liver cells (Ctrl), the cells treated with 4.5% ethanol for 5 hours when cells exhibited hallmarks of apoptosis (R0), and the treated cells that were then washed and cultured in fresh medium for 3 (R3), 6 (R6), 24 (R24) and 48 (R48) hours. Apoptosis was confirmed in the ethanol-treated cells (R0), which displayed hallmarks of apoptosis, including plasma membrane blebbing, cell shrinkage, cleavage of caspase-3 and its substrates, such as PARP and ICAD ( Figure 1A and B, images reprinted with permission ^30^). The features of apoptosis vanished after removal of the cell death inducer (R24), indicating recovery of the cells ( Figure 1A and B). Three biological replicates were performed at each time point. The principal component analysis indicated that all three biological replicates of each time point exhibited a very high correlation, as indicated by clustering, for the dataset of all 18 samples ( Figure 2A; Supplementary Figure 1; see Data availability). The unsupervised hierarchical clustering confirms the similarity between all the replicates at each time point ( Figure 2B; see Data availability; Supplementary Figure 2).

**Figure 3.**
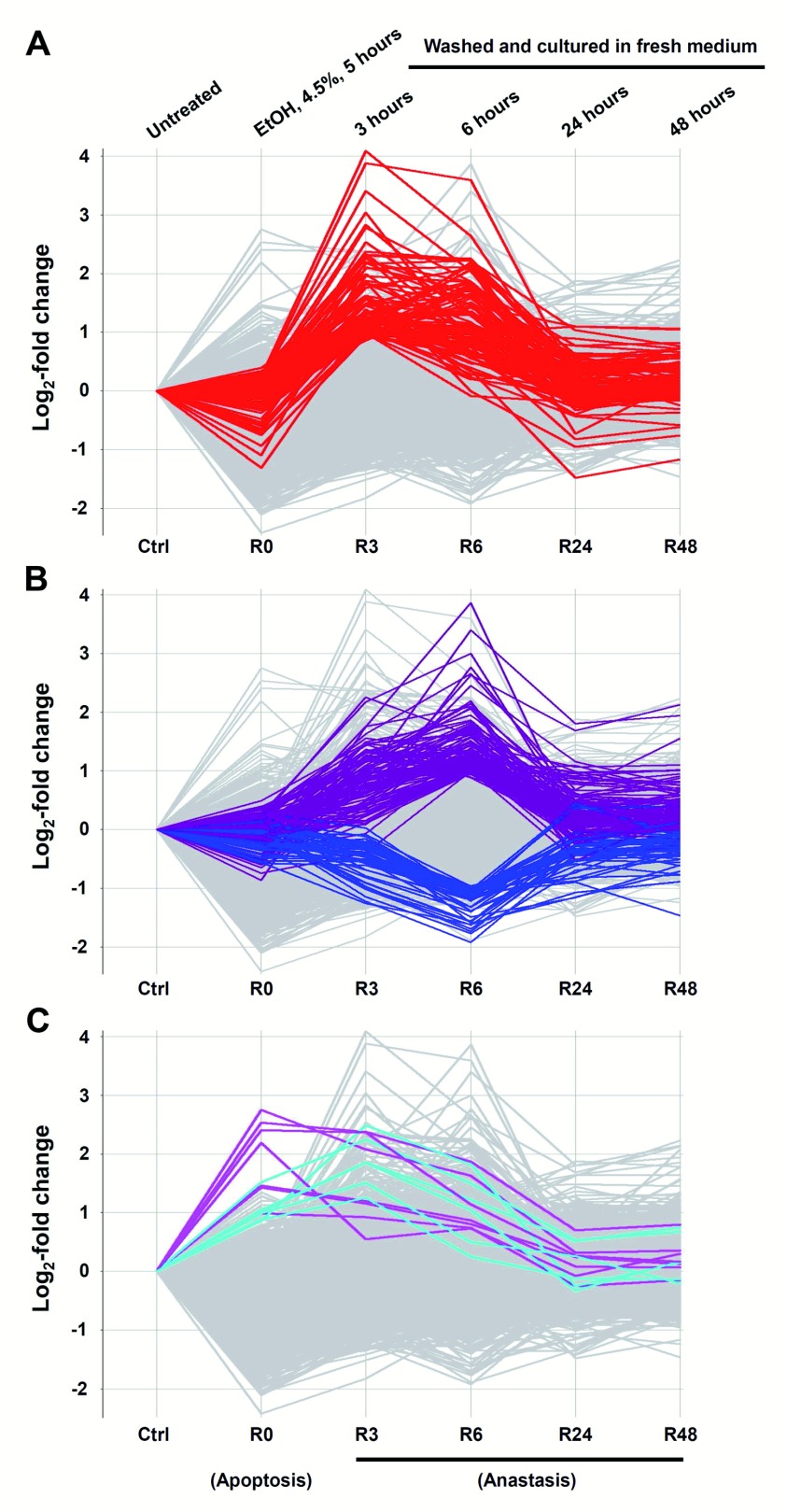
Change of gene expression profiles during reversal of apoptosis in mouse primary liver cells. Log _2_-fold change of gene expression comparison between untreated cells (Ctrl), ethanol-induced apoptotic cells (R0), and induced cells that were then washed and further cultured in fresh medium for 3 (R3), 6 (R6), 24 (R24), and 48 (R48) hours. Genes that displayed specific ( **A**) up-regulation at R3, ( **B**) up- or down-regulation at R6, and ( **C**) up-regulation anytime during the period from R0 to R6 with absolute log _2_ fold change >1 are highlighted. The log _2_ signal values from three biological replicates were averaged (geometric mean) for each time point.

Genes that display significant changes in expression during anastasis at the earliest time point of 3 hours, following the removal of the cell death inducer, may represent critical first responders of anastasis ( Figure 3A, Table 1), including transcription factors of the activator protein-1 (AP-1) family (Atf3, Fos, Fosb, Jun, Junb), transforming growth factor-β (TGF-β) signal pathway and its related regulators (Inhba, Snai1, Tgif1, Sox4, Sox9, Klf4, Klf6, Klf9), pro-survival Bcl-2 family member (Bag3), inhibitor of p53 (Mdm2), anti-oxidation (Hmox1), anti-proliferation (Btg1), DNA damage (Ddit3, Ddit4), vesicular trafficking (Vps37b) and stress-inducible (Dnajb1, Dnajb9, Herpud1, Hspb1, Hspa1b) responses. Starting at 6 hours of anastasis, other groups of gene pathways display the peak of transcription, such as cell cycle arrest (Cdkn1a, Trp53inp1), autophagy (Atg12, Sqstm1), and cell migration (Mmp9, Mmp10 and Mmp13) ( Figure 3B, Table 1 and Table 2). Expression of potent angiogenic factors, such as Vegfa and Angptl4, are up-regulated at 3 and 6 hours of anastasis, respectively ( Table 1 and Table 2). Histones display up- (Hist1h2ae, H2afj) and down- (Hist1h2ak, Hist1h2ag, Hist1h2ap, Hist1h2af, Hist2h2ac, Hist1h2ah) regulations during the first 6 hours of anastasis ( Table 2 and Table 3). Changes in expression of most of these genes peak at the 3–6-hour time points after removal of the apoptotic stimulus and then return to baseline ( Figure 3A and B; Supplementary Figure 2). Interestingly, certain genes such as splicing of pre-mRNA (Rnu6), and growth arrest and DNA repair (Gadd45g) stay up-regulated during both apoptosis and anastasis ( Figure 3C, Table 4).

**Table 1.** List of top 67 up-regulated genes at 3 ^rd^ hour (R3) of anastasis, with log _2_ fold change >1, compared with Ctrl (untreated cells).

Sort Order	Gene Symbol	Definition	Accession	Log _2_ fold change R3 vs. Ctrl
1	Atf3	activating transcription factor 3	NM_007498.2	4.08867
2	Hspa1b	heat shock protein 1B	NM_010478.2	3.88264
3	Fosb	FBJ osteosarcoma oncogene B	NM_008036.2	3.40725
4	Fos	FBJ osteosarcoma oncogene	NM_010234.2	3.03649
5	Egr2	no definition	NM_010118.1	2.82862
6	Dnajb1	DnaJ (Hsp40) homolog, subfamily B, member 1	NM_018808.1	2.78017
7	Dusp1	dual specificity phosphatase 1	NM_013642.2	2.533
8	Sox9	SRY-box containing gene 9	NM_011448.2	2.37421
9	Zfp36	zinc finger protein 36	NM_011756.4	2.33651
10	Mfsd11	no definition	AK007898	2.31434
11	Hspb1	no definition	NM_013560	2.30989
12	Jun	Jun oncogene	NM_010591.1	2.28214
13	Ddit4	DNA-damage-inducible transcript 4	NM_029083.1	2.25327
14	Vegfa	vascular endothelial growth factor A (Vegfa), transcript variant 1	NM_001025250.2	2.19637
15	Herpud1	homocysteine-inducible, ER stress-inducible, ubiquitin-like domain member 1	NM_022331.1	2.17913
16	Ddit3	DNA-damage inducible transcript 3	NM_007837.2	2.16334
17	Mdm2	transformed mouse 3T3 cell double minute 2	NM_010786.3	2.11273
18	Chac1	ChaC, cation transport regulator-like 1	NM_026929.3	2.08317
19	Arc	activity regulated cytoskeletal-associated protein	NM_018790.2	1.99046
20	Dnajb9	DnaJ (Hsp40) homolog, subfamily B, member 9	NM_013760.4	1.961
21	Zfand2a	zinc finger, AN1-type domain 2A	NM_133349.2	1.8778
22	Hes1	hairy and enhancer of split 1	NM_008235.2	1.85536
23	Bag3	BCL2-associated athanogene 3	NM_013863.4	1.85303
24	LOC100048331	PREDICTED: similar to DnaJ (Hsp40) homolog, subfamily A, member 4	XR_034509.1	1.82115
25	Hmox1	heme oxygenase	NM_010442.1	1.82111
26	Hspa5	heat shock protein 5	NM_022310.2	1.8205
27	Dlx2	distal-less homeobox 2	NM_010054.1	1.62035
28	6430590I03Rik	no definition	XM_489535	1.61804
29	Junb	Jun-B oncogene (Junb)	NM_008416.1	1.61245
30	LOC381140	no definition	XM_355056.1	1.57312
31	5430411C19Rik	PREDICTED: RIKEN cDNA 5430411C19 gene	XM_001478639.1	1.56805
32	Hspa1a	no definition	NM_010479	1.56028
33	Csrnp1	AXIN1 up-regulated 1	NM_153287.3	1.46632
34	Tnfaip3	tumor necrosis factor, alpha-induced protein 3	NM_009397.2	1.45772
35	LOC100048105	PREDICTED: similar to Ubc protein, transcript variant 1	XM_001479832.1	1.45617
36	Bhlhe40	basic helix-loop-helix domain containing, class B2	NM_011498.4	1.39137
37	Dyrk3	dual-specificity tyrosine-(Y)-phosphorylation regulated kinase 3	NM_145508.2	1.3612
38	Egr1	early growth response 1	NM_007913.5	1.35873
39	Klf9	PREDICTED: RIKEN cDNA 2310051E17 gene	XM_001479552.1	1.35306
40	Snai1	snail homolog 1	NM_011427.2	1.35105
41	Dusp2	dual specificity phosphatase 2	NM_010090.2	1.34955
42	Ubg	no definition	no accession	1.3258
43	BC022687	cDNA sequence BC022687	NM_145450.3	1.31366
44	Btg1	B-cell translocation gene 1, anti-proliferative	NM_007569.1	1.2996
45	LOC100046232	PREDICTED: similar to NFIL3/E4BP4 transcription factor	XM_001475817.1	1.27509
46	Hsph1	no definition	NM_013559.1	1.2662
47	Hist1h2ae	histone cluster 1, H2ae	NM_178187.3	1.26359
48	mtDNA_ND4L	no definition	no accession	1.2474
49	Dnajb4	DnaJ (Hsp40) homolog, subfamily B, member 4	NM_025926.1	1.24227
50	Klf4	Kruppel-like factor 4	NM_010637.1	1.23324
51	Tgif1	TGFB-induced factor homeobox 1	NM_009372.2	1.22645
52	Klf6	Kruppel-like factor 6	NM_011803.2	1.22027
53	Ppp1r10	protein phosphatase 1, regulatory subunit 10	NM_175934.2	1.21047
54	Gm16516	no definition	NM_025293.1	1.20916
55	Ifrd1	interferon-related developmental regulator 1	NM_013562.1	1.19232
56	Slc23a3	solute carrier family 23 (nucleobase transporters), member 3	NM_194333.3	1.18765
57	Mfsd11	major facilitator superfamily domain containing 11	NM_178620.3	1.16606
58	Gm4589	PREDICTED: hypothetical protein LOC100045678	XM_001475512.1	1.16498
59	Klf9	Kruppel-like factor 9	NM_010638.4	1.12553
60	Siah2	seven in absentia 2	NM_009174.3	1.11181
61	Map1lc3b	microtubule-associated protein 1 light chain 3 beta	NM_026160.3	1.10454
62	Plk2	polo-like kinase 2	NM_152804.1	1.05963
63	Fgf21	fibroblast growth factor 21	NM_020013.4	1.05538
64	Id4	inhibitor of DNA binding 4	NM_031166.2	1.04488
65	Csf1	colony stimulating factor 1	NM_007778.3	1.03533
66	Bbc3	BCL2 binding component 3 (Bbc3)	NM_133234.1	1.03288
67	6230400G14Rik	no definition	no accession	1.02327

**Table 2.** List of top 109 up-regulated genes at 6 ^th^ hour (R6) of anastasis, with log _2_ fold change >0.93, compared with Ctrl (untreated cells).

Sort Order	Gene Symbol	Definition	Accession	Log _2_ fold change R6 vs. Ctrl
1	Inhba	inhibin beta-A	NM_008380.1	3.86584
2	Mmp10	matrix metallopeptidase 10	NM_019471.2	3.39644
3	Lce1f	late cornified envelope 1F	NM_026394.2	2.99957
4	Serpinb2	serine (or cysteine) peptidase inhibitor, clade B, member 2	NM_011111.3	2.77022
5	Serpina3h	serine (or cysteine) peptidase inhibitor, clade A, member 3H	NM_001034870.2	2.65107
6	Mmp13	matrix metallopeptidase 13	NM_008607.1	2.62637
7	Ptpn22	protein tyrosine phosphatase, non-receptor type 22	NM_008979.1	2.45292
8	Rgs16	regulator of G-protein signaling 16	NM_011267.2	2.18647
9	Nppb	natriuretic peptide precursor type B	NM_008726.3	2.15071
10	Has1	hyaluronan synthase1	NM_008215.1	2.14235
11	Dusp5	no definition	XM_140740.3	2.09536
12	Sqstm1	sequestosome 1	NM_011018.2	2.07477
13	Nupr1	nuclear protein 1	NM_019738.1	2.06313
14	Sphk1	sphingosine kinase 1 (Sphk1), transcript variant 2	NM_025367.5	1.94856
15	Dusp4	dual specificity phosphatase 4	NM_176933.4	1.85742
16	Klhl21	kelch-like 21	NM_001033352.3	1.84531
17	Lor	loricrin	NM_008508.2	1.81763
18	Ndrg1	N-myc downstream regulated gene 1	NM_008681.2	1.79158
19	Srxn1	sulfiredoxin 1 homolog	NM_029688.4	1.78335
20	Hk2	PREDICTED: hypothetical protein LOC100047934	XM_001478074.1	1.7519
21	Txnrd1	thioredoxin reductase 1 (Txnrd1), transcript variant 1	NM_001042523.1	1.75148
22	Angptl4	no definition	NM_020581	1.72982
23	Trib3	tribbles homolog 3	NM_175093.2	1.72246
24	C330006P03Rik	no definition	no accession	1.71297
25	Cdkn1a	cyclin-dependent kinase inhibitor 1A	NM_007669.2	1.69118
26	Gdf15	growth differentiation factor 15	NM_011819.1	1.67887
27	Prkg2	protein kinase, cGMP-dependent, type II	NM_008926.3	1.67374
28	H2afj	H2A histone family, member J	NM_177688.2	1.64825
29	Hbegf	heparin-binding EGF-like growth factor	NM_010415.1	1.61893
30	Trp53inp1	transformation related protein 53 inducible nuclear protein 1	NM_021897.1	1.61348
31	Gfpt2	glutamine fructose-6-phosphate transaminase 2	NM_013529.2	1.58159
32	Slc7a11	no definition	AK037490	1.57761
33	Ndrg1	no definition	NM_008681	1.5652
34	Gprc5a	G protein-coupled receptor, family C, group 5, member A	NM_181444.3	1.51339
35	Ibrdc3	no definition	XM_204030	1.49816
36	Ngf	nerve growth factor, beta	NM_013609.1	1.48619
37	Lce1d	late cornified envelope 1D	NM_027137.2	1.44977
38	Tpsab1	tryptase alpha/beta 1	NM_031187.2	1.44267
39	Htr2b	5-hydroxytryptamine (serotonin) receptor 2B	NM_008311.2	1.43265
40	Sox4	SRY-box containing gene 4	NM_009238.2	1.41763
41	Il1rl1	interleukin 1 receptor-like 1 (Il1rl1), transcript variant 1	NM_001025602.1	1.3994
42	Prr9	RIKEN cDNA A030004J04 gene (A030004J04Rik)	NM_175424.3	1.36416
43	Vgf	VGF nerve growth factor inducible	NM_001039385.1	1.35246
44	Errfi1	ERBB receptor feedback inhibitor 1	NM_133753.1	1.34582
45	Il6	interleukin 6	NM_031168.1	1.33283
46	Gprc5a	no definition	NM_181444	1.31955
47	Antxr2	anthrax toxin receptor 2	NM_133738.1	1.30719
48	Tgif1	TGFB-induced factor homeobox 1	NM_009372.2	1.29814
49	Krt8	keratin 8	NM_031170.2	1.28819
50	2300009A05Rik	PREDICTED: RIKEN cDNA 2300009A05 gene, transcript variant 3	XM_898537.2	1.26684
51	Dppa5a	developmental pluripotency associated 5	NM_025274.1	1.258
52	Mt2	metallothionein 2	NM_008630.2	1.2441
53	Plaur	plasminogen activator, urokinase receptor	NM_011113.3	1.22553
54	Thbd	thrombomodulin	NM_009378.2	1.22252
55	LOC100047353	PREDICTED: similar to myocardial vascular inhibition factor	XM_001477963.1	1.22053
56	Csf2	colony stimulating factor 2 (granulocyte-macrophage)	NM_009969.4	1.22019
57	Map2k1	mitogen-activated protein kinase kinase 1	NM_008927.3	1.21788
58	Dpp7	dipeptidylpeptidase 7	NM_031843.2	1.21624
59	LOC672274	PREDICTED: similar to Transcription factor SOX-4	XR_003788.1	1.21149
60	Blcap	bladder cancer associated protein homolog	NM_016916.3	1.21046
61	Zfc3h1	no definition	NM_001033261.2	1.20585
62	Dusp6	dual specificity phosphatase 6	NM_026268.1	1.20441
63	Areg	amphiregulin	NM_009704.3	1.19656
64	C630022N07Rik	no definition	no accession	1.19569
65	Denr	density-regulated protein	NM_026603.1	1.18464
66	Slc3a2	solute carrier family 3 (activators of dibasic and neutral amino acid transport), member 2	NM_008577.3	1.18244
67	Ern1	endoplasmic reticulum (ER) to nucleus signalling 1	NM_023913.2	1.15145
68	Dnmt3l	DNA (cytosine-5-)-methyltransferase 3-like (Dnmt3l), transcript variant 2	NM_001081695.1	1.13992
69	D130007C19Rik	no definition	AK051152	1.13724
70	LOC100046401	PREDICTED: similar to SDR2	XR_032583.1	1.1332
71	Sh3bp2	SH3-domain binding protein 2	NM_011893.2	1.11999
72	Tgoln1	trans-golgi network protein	NM_009443.3	1.11454
73	Gm12226	similar to oxidative stress responsive 1 (Rp23-297j14.5)	NM_001099322.1	1.11231
74	Stk40	no definition	NM_028800	1.11149
75	Marcksl1	MARCKS-like 1 (Marcksl1), mRNA.	NM_010807.3	1.09791
76	Ypel5	yippee-like 5 (Drosophila) (Ypel5), mRNA.	NM_027166.3	1.08882
77	Fam180a	No definition	NM_173375	1.08779
78	Creb3l2	cAMP responsive element binding protein 3-like 2 (Creb3l2), mRNA.	NM_178661.3	1.08689
79	Ly96	lymphocyte antigen 96 (Ly96), mRNA.	NM_016923.1	1.06285
80	Igf2bp2	insulin-like growth factor 2 mRNA binding protein 2 (Igf2bp2), mRNA.	NM_183029.1	1.06145
81	Mafg	v-maf musculoaponeurotic fibrosarcoma oncogene family, protein G	NM_010756.3	1.05594
82	Cttnbp2nl	No definition	NM_030249	1.04697
83	Col20a1	PREDICTED: collagen, type XX, alpha 1 (Col20a1), mRNA.	XM_181390.5	1.04143
84	Vps37b	vacuolar protein sorting 37B (yeast) (Vps37b), mRNA.	NM_177876.4	1.03812
85	A530046M15	No definition	XM_488663	1.03773
86	Eid3	EP300 interacting inhibitor of differentiation 3 (Eid3), mRNA.	NM_025499.2	1.03567
87	Nabp1	oligonucleotide/oligosaccharide-binding fold containing 2A (Obfc2a), mRNA.	NM_028696.2	1.0351
88	Pqlc1	PQ loop repeat containing 1 (Pqlc1), mRNA.	NM_025861.2	1.03363
89	Whrn	whirlin (Whrn), transcript variant 6, mRNA.	NM_001008795.1	1.0255
90	Cish	cytokine inducible SH2-containing protein (Cish), mRNA.	NM_009895.3	1.02328
91	Ptpre	protein tyrosine phosphatase, receptor type, E (Ptpre), mRNA.	NM_011212.2	1.01915
92	Bach1	BTB and CNC homology 1 (Bach1), mRNA.	NM_007520.2	1.01808
93	Cyb5r1	cytochrome b5 reductase 1 (Cyb5r1), mRNA.	NM_028057.2	1.01401
94	Slc1a4	solute carrier family 1 (glutamate/neutral amino acid transporter), member 4	NM_018861.2	1.00471
95	Mmd	no definition	AK033889	0.998067
96	Slc6a9	solute carrier family 6 (neurotransmitter transporter, glycine), member 9	NM_008135.4	0.994683
97	LOC100047963	PREDICTED: similar to ADIR1	XM_001479238.1	0.994667
98	Atf4	activating transcription factor 4	NM_009716.2	0.982833
99	Cttnbp2nl	CTTNBP2 N-terminal like	NM_030249.3	0.970113
100	Mmp9	matrix metallopeptidase 9	NM_013599.2	0.968853
101	Hmga1	high mobility group AT-hook 1	NM_016660.2	0.96846
102	Phlda1	pleckstrin homology-like domain, family A, member 1	NM_009344.1	0.963867
103	Aars	alanyl-tRNA synthetase	NM_146217.3	0.962397
104	Angpt2	angiopoietin 2	NM_007426.3	0.95926
105	Zswim4	zinc finger, SWIM domain containing 4	NM_172503.3	0.957373
106	Selk	no definition	NM_019979.1	0.954917
107	Abhd2	abhydrolase domain containing 2	NM_018811.6	0.954587
108	Krtap4-16	predicted gene, OTTMUSG00000002196	NM_001013823.1	0.95438
109	Atg12	autophagy-related 12	NM_026217.1	0.94998

**Table 3.** List of top 50 down-regulated genes at 6 ^th^ hour (R6) of anastasis, with log _2_ fold change <-0.95, compared with Ctrl (untreated cells).

Sort Order	Gene Symbol	Definition	Accession	Log _2_ fold change R6 vs. Ctrl
1	Hist1h2ak	histone cluster 1, H2ak	NM_178183.1	-1.91761
2	Hist1h2ag	histone cluster 1, H2ag	NM_178186.2	-1.76767
3	Hist1h2ap	histone cluster 1, H2ao	NM_178185.1	-1.7396
4	Hist1h2af	histone cluster 1, H2af	NM_175661.1	-1.6854
5	Hist2h2ac	histone cluster 2, H2ac	NM_175662.1	-1.6272
6	Slc1a3	solute carrier family 1 (glial high affinity glutamate transporter), member 3	NM_148938.2	-1.61827
7	9930013L23Rik	no definition	AK018112	-1.59264
8	Hist1h2ah	histone cluster 1, H2ah	NM_175659.1	-1.57002
9	Hist1h2al	PREDICTED: predicted gene, EG667728	XR_035278.1	-1.56907
10	Hist1h2ad	histone cluster 1, H2ad	NM_178188.3	-1.56233
11	Scel	sciellin	NM_022886.2	-1.48845
12	Hist1h2ai	histone cluster 1, H2ai	NM_178182.1	-1.40187
13	Fzd2	frizzled homolog 2	NM_020510.2	-1.38203
14	Sdpr	serum deprivation response	NM_138741.1	-1.38033
15	Hs3st1	heparan sulfate (glucosamine) 3-O-sulfotransferase 1	NM_010474.1	-1.32418
16	Hist2h2ab	histone cluster 2, H2ab	NM_178213.3	-1.30977
17	Kif2c	kinesin family member 2C (Kif2c) XM_986361	NM_134471.3	-1.21821
18	Fam198b	RIKEN cDNA 1110032E23 gene (1110032E23Rik)	NM_133187.2	-1.1988
19	Cdc42ep2	CDC42 effector protein (Rho GTPase binding) 2	NM_026772.1	-1.19681
20	Lurap1l	DNA segment, Chr 4, Brigham & Women's Genetics 0951 expressed (D4Bwg0951e)	NM_026821.4	-1.18656
21	Medag	RIKEN cDNA 6330406I15 gene	NM_027519.1	-1.18243
22	Disp1	dispatched homolog 1	NM_026866.2	-1.18107
23	Bmp4	bone morphogenetic protein 4	NM_007554.2	-1.16637
24	Rab27a	RAB27A, member RAS oncogene family	NM_023635.4	-1.13917
25	Aurka	aurora kinase A	NM_011497.3	-1.12507
26	Ncaph	non-SMC condensin I complex, subunit H	NM_144818.1	-1.12132
27	Fignl1	fidgetin-like 1	NM_021891.2	-1.10521
28	Dbp	D site albumin promoter binding protein	NM_016974.1	-1.09945
29	Meis2	Meis homeobox 2 (Meis2), transcript variant 2	NM_010825.2	-1.08487
30	Synpo	PREDICTED: synaptopodin, transcript variant 2	XM_981156.1	-1.08076
31	Hist1h2an	histone cluster 1, H2an	NM_178184.1	-1.0804
32	Fam111a	RIKEN cDNA 4632417K18 gene (4632417K18Rik)	NM_026640.2	-1.07617
33	Aurkb	aurora kinase B	NM_011496.1	-1.07507
34	Anln	anillin, actin binding protein	NM_028390.2	-1.07218
35	Tuft1	tuftelin 1	NM_011656.2	-1.06969
36	Cxcl12	chemokine (C-X-C motif) ligand 12 (Cxcl12), transcript variant 1	NM_021704.2	-1.0664
37	Sipa1l1	signal-induced proliferation-associated 1 like 1	NM_172579.1	-1.03567
38	Rbms2	RNA binding motif, single stranded interacting protein 2	NM_019711.2	-1.03096
39	Wdr6	WD repeat domain 6	NM_031392.2	-1.02705
40	Tk1	thymidine kinase 1	NM_009387.1	-1.02669
41	Mylk	myosin, light polypeptide kinase	NM_139300.3	-1.01621
42	Slc9a3r1	solute carrier family 9 (sodium/hydrogen exchanger), member 3 regulator 1	NM_012030.2	-1.0137
43	Kif22	kinesin family member 22	NM_145588.1	-1.01346
44	Speer3	spermatogenesis associated glutamate (E)-rich protein 3	NM_027650.2	-1.01229
45	Mrgprf	MAS-related GPR, member F	NM_145379.2	-1.01038
46	Bub1b	budding uninhibited by benzimidazoles 1 homolog, beta	NM_009773.1	-1.00547
47	Pcgf5	polycomb group ring finger 5	NM_029508.3	-1.00513
48	Marcks	myristoylated alanine rich protein kinase C substrate	NM_008538.2	-0.973133
49	Fam83d	2310007D09Rik	NM_027975.1	-0.966323
50	Slc16a4	solute carrier family 16 (monocarboxylic acid transporters), member 4	NM_146136.1	-0.96461

**Table 4.** List of top 15 up-regulated genes during apoptosis (R0) and anastasis (R3 and R6), with log _2_ fold change >1 either on R0, R3, or R6, compared with Ctrl (untreated cells).

				Log _2_ fold change			
Sort Order	Gene Symbol	Definition	Accession	R0 vs. Ctrl	R3 vs. Ctrl	R6 vs. Ctrl	R24 vs. Ctrl
1	Rnu6	U6 small nuclear RNA	NR_003027.1	2.75163	2.08117	1.63203	0.315967
2	Med23	no definition	AK042346	2.53792	2.37555	1.87041	0.70258
3	Prf1	perforin 1	NM_011073.2	2.40981	2.36444	1.1381	0.262567
4	F830002E14Rik	no definition	AK089567	2.18787	0.549207	0.731387	‐0.08211
5	Slc11a1	solute carrier family 11 (proton-coupled divalent metal ion transporters), member 1	NM_013612.1	1.51837	2.24547	1.50337	0.53101
6	Hist1h4a	histone cluster 1, H4a	NM_178192.1	1.46352	1.19978	0.87087	0.08441
7	Hist1h4j	histone cluster 1, H4j	NM_178210.1	1.4276	1.15198	0.801933	0.241233
8	2310005L22Rik	no definition	no accession	1.19244	1.23574	0.79319	0.0878833
9	2810026P18Rik	no definition	no accession	1.12393	1.14743	0.527953	‐0.31585
10	Gadd45g	growth arrest and DNA-damage- inducible 45 gamma	NM_011817.1	1.04177	1.85013	1.0444	‐0.324567
11	Sppl3	no definition	AK047886	1.01269	1.85284	1.22205	0.51878
12	1810026B05Rik	no definition	XM_489186	0.9892	0.92441	0.742947	‐0.257843
13	BC030476	cDNA sequence BC030476	NM_173421.1	0.98391	1.51116	0.495447	0.2612
14	Zbtb2	zinc finger and BTB domain containing 2	NM_001033466.1	0.882457	1.25171	0.253943	‐0.116403
15	Ppp1r15a	myeloid differentiation primary response gene 116	NM_008654.1	0.862993	2.48696	1.82011	‐0.25323

**Figure 4.**
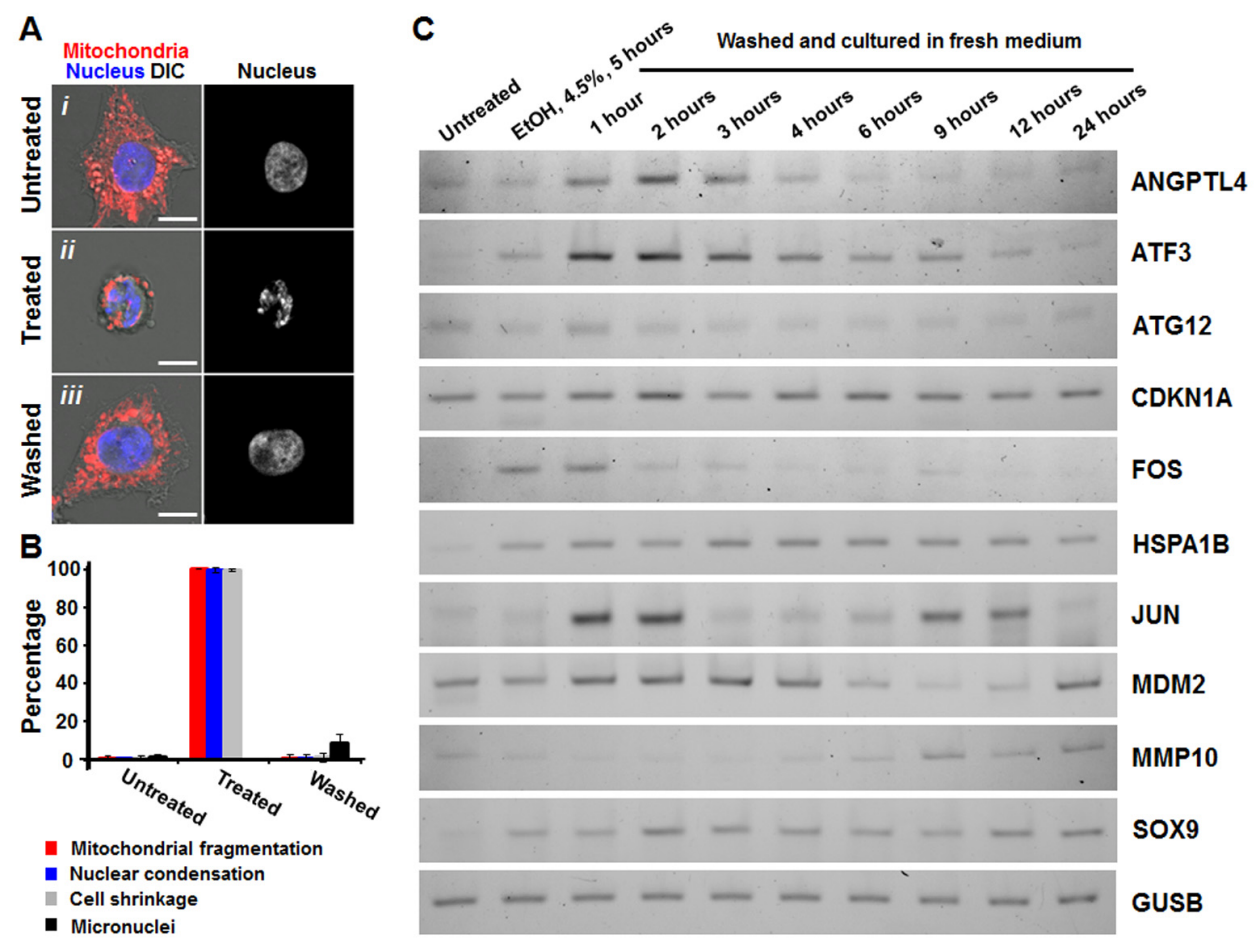
Change of gene expressions during reversal of apoptosis in human liver cancer HepG2 cells. ( **A**) Confocal and differential interference contrast (DIC) microscopy of untreated liver cells ( ***i*** Untreated), cells that were exposed to 4.5% ethanol for 5 hours ( ***ii*** Treated), and the treated cells that were washed to remove apoptosis inducer and further cultured for 6 hours ( ***iii*** Washed). Merged images, mitochondria (red) and nuclei (blue) were visualized by confocal microscopy and cell morphology by DIC. Monochrome images, nucleus of the corresponding cells. Scale bar, 10 μm. ( **B**) Quantification of the apoptotic response and its reversal on HepG2 cells. Percentage of the untreated cells, the treated cells (4.5% ethanol, 5 hours) and the washed cells (24 hours) showing mitochondrial fragmentation, nuclear condensation, cell shrinkage, and formation of micronuclei. ( **C**) RT-PCR gel analysis of changes in mRNA levels of ANGPTL4, ATF3, ATG12, CDKN1A, FOS, GUSB, HSPA1B, JUN, MDM2, MMP10 and SOX9 on the untreated (Ctrl), the treated (R0, 4.5% ethanol for 5 hours), and the treated cells that were then washed and incubated in fresh medium for 1 hour (R1), 2 hours (R2), 3 hours (R3), 4 hours (R4), 6 hours (R6), 9 hours (R9), 12 hours (R12), and 24 hours (R24). GUSB serves as housekeeping gene. Sequences of primer sets for detecting targeted genes are available in Table 5.

We further observed the similar changes in gene expressions during anastasis in cultured human liver cancer HepG2 cells ( Figure 4; see Data availability). The untreated HepG2 cells displayed tubular and filamentous mitochondria in the cells that spread on the substrate ( Figure 4A *i*, 4B). After exposure to 4.5% ethanol for 5 hours, the treated cells displayed morphological hallmarks of apoptosis, such as mitochondrial fragmentation, nuclear condensation, plasma membrane blebbing, and cell shrinkage ( Figure 4A *ii*, 4B). After washed and incubated with fresh culture medium, the treated cells regained normal morphology ( Figure 4A *iii*, 4B). Interestingly, the HepG2 cells that underwent anastasis displayed the increase in micronuclei formation ( Figure 4B), which is the biomarker of DNA damage ^58^, as we previously observed in mouse primary liver cells, mouse embryonic fibroblast NIH 3T3 cells, human cervical cancer HeLa cells, and human small cell lung carcinoma H446 cells ^30, 31^. By using reverse transcription polymerase chain reaction (RT-PCR), we verified our microarray data on HepG2 cells during reversal of ethanol-induced apoptosis, and found similar gene expression patterns during anastasis, including changes in mRNA levels of ANGPTL4, ATF3, ATG12, CDKN1A, FOS, HSPA1B, JUN, MDM2, MMP10 and SOX9 ( Figure 4C; Supplementary Figure 3). This suggests that the mechanism of anastasis is conserved between primary and cancer cells.

**Table 5.** List of primer sequences for RT-PCR.

Gene	Accession number	Forward primer	Reverse primer	Amplicon
ANGPTL4	NM_139314.2	gacaagaactgcgccaaga	gccgttgaggttggaatg	72
ATF3	NM_001674.3	cgtgagtcctcggtgctc	gcctgggtgttgaagcat	112
ATG12	NM_004707.3	tcttccgctgcagtttcc	gtctcccacagcctttagca	87
CDKN1A	NM_000389.4	tgggtggtaccctctgga	tgaatttcataaccgcctgtg	65
FOS	NM_005252	ctggcgttgtgaagaccat	ccttttctcttcttcttctggagat	95
GUSB	NM_000181.3	cgccctgcctatctgtattc	tccccacagggagtgtgtag	91
HSPA1B	NM_005346.4	gggtcaggccctaccatt	caacagtccacctcaaagacaa	77
JUN	NM_002228.3	ccaaaggatagtgcgatgttt	ctgtccctctccactgcaac	62
MDM2	NM_002392.5	tctgatagtatttccctttcctttg	tgttcacttacaccagcatcaa	137
MMP10	NM_002425.2	gcattttggccctctcttc	cagggtatggatgcctcttg	147
SOX9	NM_000346.3	gtacccgcacttgcacaac	tctcgctctcgttcagaagtc	74

**Figure 5.**
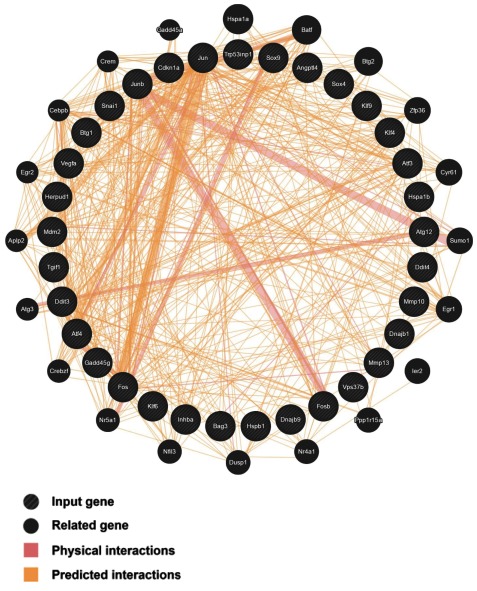
Interaction network of the up-regulated genes during anastasis. The 33 up-regulated genes during anastasis were selected for analysis using GeneMANIA.

The change in transcriptional profiles during anastasis provides us mechanistic insights into how dying cells could reverse apoptosis ( Figure 5). In early anastasis, our data reveals that the regulators of the TGF-β signalling pathway, which control various fundamental cellular and pathological process, including proliferation, cell survival, apoptosis, cell migration, and transformation ^59– 62^, are upregulated. The activation of the TGF-β pathway is further supported by the upregulation of AP-1 (Jun-Fos) ^59^, as observed here during early anastasis. The up-regulation of the TGF-β pathway can also promote the expression of murine double minute 2 (Mdm2) ^63, 64^, an inhibitor of p53 that is also up-regulated during early anastasis ^30^. As p53 plays a critical role in regulating apoptosis and DNA repair ^65, 66^, the expression of Mdm2 could not only promote cell survival by inhibiting p53-mediated cell death, but also cause mutations as we have observed in the cells after anastasis ^30^. Expression of Mdm2 can also activate XIAP ^67^, which inhibits caspases 3, 7 and 9 ^68– 73^, and therefore, could promote anastasis by suppressing the caspase-mediated cell destruction process. Up-regulation of anti-apoptotic BCL2 protein (Bag3) and heat shock proteins (Hsps) during anastasis can also neutralize pro-apoptotic proteins to promote cell recovery ^26, 74, 75^. Expression of Hmox1, which encodes heme oxygenase ^76^, could protect dying cells from free radicals that are generated during apoptosis. Notably, the expression of Bbc3, a pro-apoptotic BH3-only gene to encode PUMA (p53 upregulated modulator of apoptosis) ^77, 78^, peaks at anastasis (R3-R6), suggesting the sign of anastasis vs apoptosis in the recovering cells during the early stage of the cell recovery process.

To reverse apoptosis, the recovering cells need to remove or recycle the destroyed cellular components, such as the toxic or damaged proteins that are cleaved by caspases, and dysfunctional organelles like the permeabilized mitochondria. Autophagy could contribute to anastasis, as the recovering cells display up-regulation of Atg12 ( Figure 3B, Table 2), which is important to the formation of autophagosome to engulf the materials that are then transported to lysosomes or vacuoles for degradation ^79– 82^. Sqstm1, which encodes sequestosome 1 ^83– 85^ and is up-regulated at R6, could play important role in mediating autophagy and DNA damage response during anastasis. In fact, recently studies reveal that autophagy can be activated by the DNA damage response, and play a role in maintaining the nuclear and mitochondrial genomic integrity through DNA repair and removal of micronuclei and damaged nuclear parts ^86, 87^. This could suppress mutagenesis and oncogenic transformation to occur in the cells that reverse apoptosis as we have observed after DNA damage ^30, 31^. Autophagy is also implicated in the exosome secretory pathway ^88– 90^, which could allow rapid clearance of damaged or toxic materials during anastasis through exosomes. Interestingly, our microarray data shows that the recovering cells display up-regulation of potent angiogenic factors such as Vegfa and Angptl4 ( Figure 3A and B, Table 1 and Table 2), which promote vascular permeability and angiogenesis ^91– 94^. This could facilitate anastasis by supplying nutrient and clearing waste products. However, this could also enhance tumour recurrence, progression and metastasis ^95^, when anastasis occurs in cancer cells between cycles of cancer therapy. In fact, our data also reveals the up-regulation of genes involved in cell migration during anastasis ^30^, such as Mmp 9, 10 and 13 ( Figure 3B, Table 2) that encode matrix metalloproteinases ^96– 99^. This could be a stress-inducible response that promotes cell migration, like what we have observed in HeLa cells after anastasis ( Supplementary Figure 4) ^31^, which might contribute to wound healing after tissue injury, or metastasis during cancer recurrence ^100, 101^.

Change in expression of histone proteins contributes to histone modification, which plays critical role in transcription, DNA replication and repairing ^102– 106^. At the late stage of anastasis (R6), various histone genes display significant changes in expression ( Table 2, Table 3), suggesting potential connection between histone modification and reversal of apoptosis. Interestingly, significant number of histone genes are down-regulated during anastasis ( Table 3). Recent study reported histone degradation in response to DNA damage, and that is important for DNA repairing ^107^. As dying cells can reverse apoptosis after DNA damage ^30, 31^, reduction of histone gene expression could represent the DNA damage response during anastasis. 

Arresting cell cycle during anastasis is important as it can allow damaged cells to be repaired before they restore proliferation. This hypothesis is supported by our microarray data that reveals up-regulation of genes that suppress cell cycle ( Figure 3A–C). For example, B-cell translocation gene 1 (Btg1) is an anti-proliferative gene ^108, 109^, which is up-regulated during the early anastasis (R3). At later stage of anastasis (R6), other cell cycle inhibitors express, including Cdkn1a which encodes p21 that induces cell cycle arrest and senescence ^110– 112^, and also Trp53inp1 which encodes tumor protein p53-inducible nuclear protein 1 that can arrest cell cycle independent to p53 expression ^113^. These suggest that cell cycle is suppressed by multiple pathways during anastasis.

We also identified genes that are up-regulated both during apoptosis and anastasis, such as Gadd45g, and Rnu6 ( Figure 3C, Table 4). Gadd45g functions in growth arrest and DNA repair ^114, 115^, and therefore, could be the cytoprotective mechanism that preserves DNA in the dying cells during cell death induction (R0), and promotes the injured cells to repair when the environment is improved (R3 and R6). Rnu6 encodes U6 small nuclear RNA, which is important for splicing of a mammalian pre-mRNA ^116– 119^. Upregulation of Rnu6 from R0 to R6 suggests that post-transcriptional regulation could be involved during apoptosis and anastasis. In fact, translational regulation also contributes to anastasis. For example, caspase-3, PARP and ICAD are cleaved in dying cells during apoptosis, and the non-cleaved form of corresponding proteins restores after anastasis ( Figure 1B). Interestingly, the mRNA level of caspase-3, PARP and ICAD did not show significant increase during and after anastasis (see Data availability). This suggests the contribution of translational regulation during anastasis.

**Figure 6.**
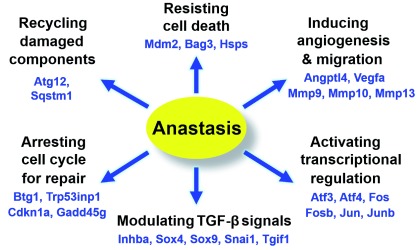
Up-regulation of genes and potential corresponding pathways during reversal of apoptosis.

**Figure 7.**
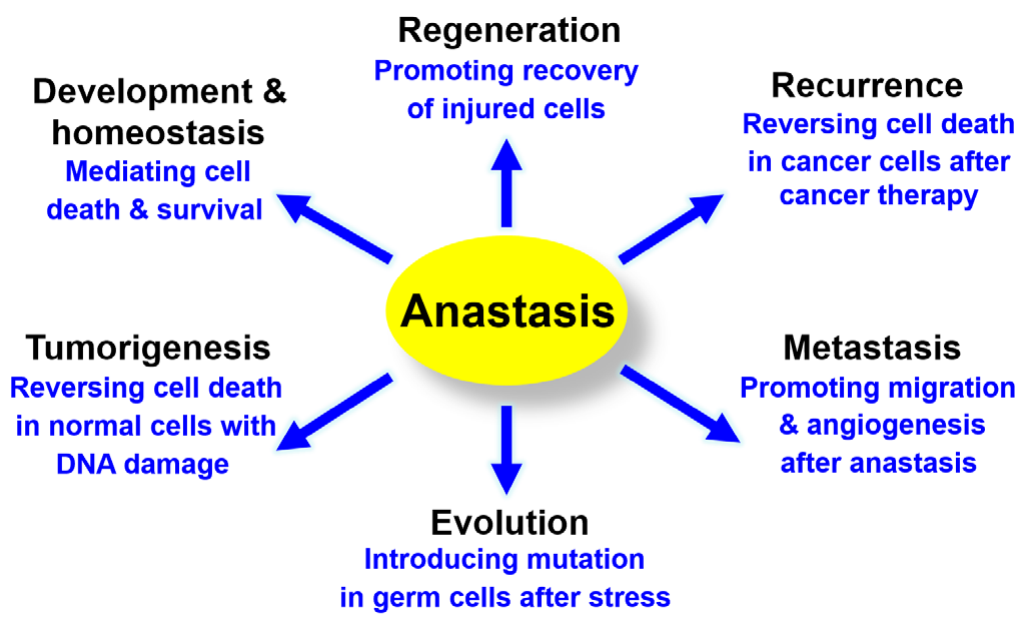
Potential consequences of anastasis.

Our study provides new insights into the mechanisms and consequences of anastasis ( Figure 6). Researchers can analyse our microarray data to further identify the hallmarks of anastasis, understand its role, elucidate molecular mechanisms that reverse apoptosis, and develop therapeutic strategies by controlling anastasis. To identify the genes that display specific change on a transcriptional level, software such as Spotfire can be used to view the gene expression pattern at different time points during the reversal of apoptosis ^54^. To study the molecular mechanism of anastasis, Ingenuity Pathway Analysis can be used to create mechanistic hypotheses according to the transcriptional profile ^120^. To identify drugs that modulate anastasis, Connectivity Map can be used to identify small molecules that promote or suppress anastasis based on its gene expression signature ^121, 122^. Anastasis could be a cell survival phenomenon mediated by multiple pathways ^29– 31, 33^, so by comparing the gene expression profiles, researchers can study its potential connection to other cellular processes, such as anti-apoptotic pathways, autophagy, and stress-inducible responses ^82, 123– 127^. By searching the molecular signature of anastasis, researchers can study its potential contribution to physiological and pathological conditions, such as recovery from heart failure, wound healing, mutagenesis, tumour evolution, cancer recurrence and metastasis ^45, 100, 101, 128^. Further data analysis will stimulate the generation of hypotheses for future studies involving anastasis. As our understanding of anastasis mechanism expands, it will uncover its potential impacts on physiology and pathology, and offer exciting new therapeutic opportunities to intractable diseases by mediating cell death and survival ( Figure 7).

## Data availability

The data referenced by this article are under copyright with the following copyright statement: Copyright: © 2017 Tang HM et al.

Data associated with the article are available under the terms of the Creative Commons Zero "No rights reserved" data waiver (CC0 1.0 Public domain dedication). 

*Figshare*: Raw data for Tang *et al.*, 2016 “Molecular signature of anastasis for reversal of apoptosis” doi: 10.6084/m9.figshare.4502732

http://dx.doi.org/10.6084/m9.figshare.4502732

## Supplementary material

**Supplementary Figure 1: Technical validation of microarray data.** The three biological replicate samples of microarray data were shown to cluster together by using principal component analysis (PCA).

**Supplementary Figure 2: Genes display unique expression patterns at each timepoint.** Output of all genes analysed by Spotfire (K cluster, also see Data availability).

**Supplementary Figure 3. Uncropped agarose gel images.** RT-PCR gel analysis of changes in mRNA levels of ANGPTL4, ATF3, ATG12, CDKN1A, FOS, HSPA1B, JUN, MDM2, MMP10 and SOX9 on untreated (Lane 1: Ctrl), treated (Lane 2: R0, 4.5% ethanol for 5 hours), and the treated cells that were then washed and incubated in fresh medium for 1 hour (Lane 3: R1), 2 hours (Lane 4: R2), 3 hours (Lane 5: R3), 4 hours (Lane 6: R4), 6 hours (Lane 7: R6), 9 hours (Lane 8: R9), 12 hours (Lane 9: R12), and 24 hours (Lane 10: R24). GUSB serves as housekeeping gene. Lane M is loaded with 25 bp DNA Ladder (Invitrogen). Sequences of primer sets for detecting targeted genes are available in Table 5.

**Supplementary Figure 4: Increase in mobility of HeLa cells after reversal of ethanol-induced apoptosis.** Time-lapse live-cell fluorescence microscopy of HeLa cells before, during, and after exposure to the apoptotic stimulus, ethanol. The same cells before ethanol induction (Untreated, *i*), induced with 5% ethanol in culture medium for 55 minutes (Induced, *ii* to *iv*), and then washed and further cultured with fresh medium (Washed, *v* to *xx*). Green, white and yellow lines indicate the footprints of the corresponding same cells that reversed apoptosis throughout the experiment with time. Cells without displayed hallmarks of apoptosis at late stage, such as cell shrinkage and nucleus condensation (without track), had lower mobility than the tracked cells. Merged images, mitochondria (red) and nuclei (blue) were visualized by fluorescence, and cell morphology by differential interference contrast (DIC) microscopy. Time presented as hour:minute. Scale bar, 10 μm. Adopted images reprinted with permission ^129^.
