# Supplementary figures and images for: Molecular signature of anastasis for reversal of apoptosis
Source: F1000Res. 2017 Feb 9;6:43. Originally published 2017 Jan 13. [Version 2] doi: 10.12688/f1000research.10568.2 (PMC5310529; doi:10.12688/f1000research.10568.2)

**
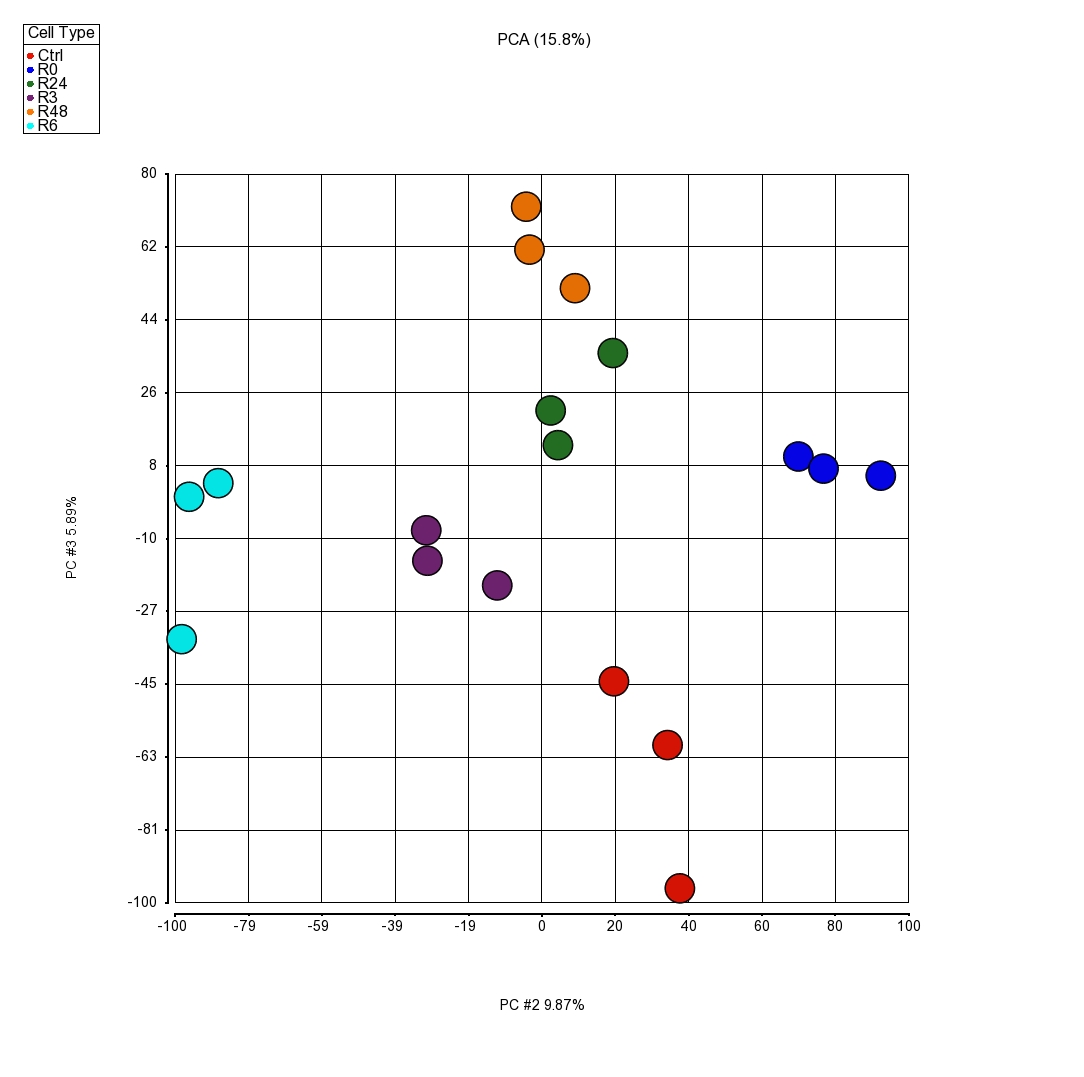
Supplementary figure 1**

**Supplementary figure 2**

**
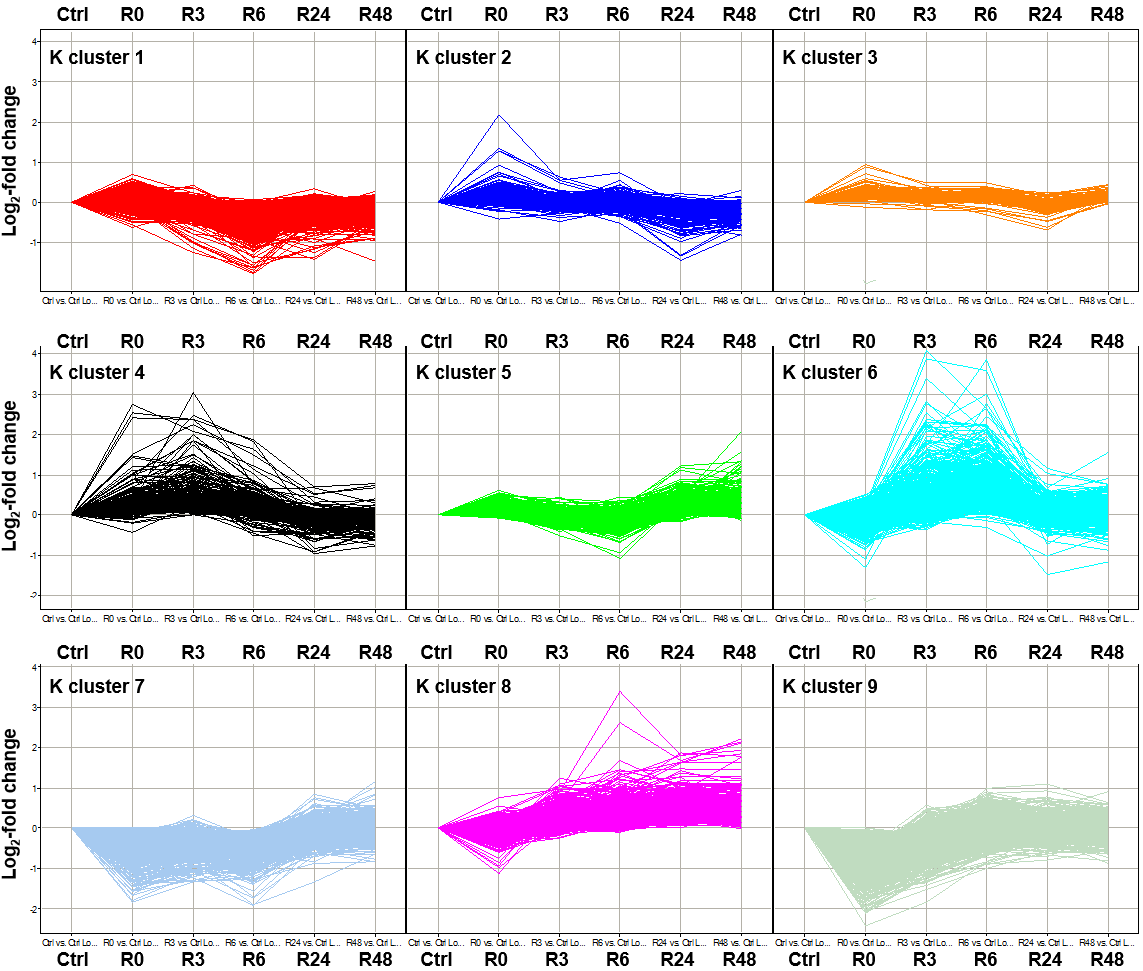
**

**
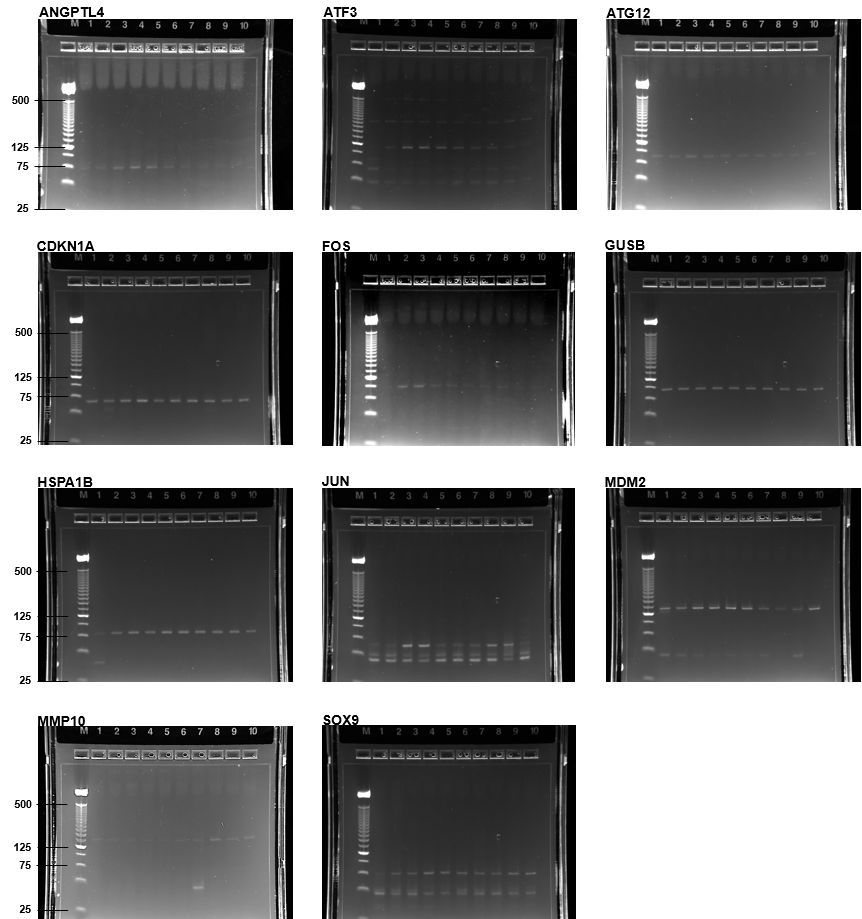
Supplementary figure 3**

**Supplementary figure 4**

Supplement: Supplementary file 1 [file f1000research-6-11669-s0000.tgz › a2e756f7-d0ed-45b2-866a-b3a29a6363a5.docx]
