# Supplementary material for: Molecular signature of anastasis for reversal of apoptosis
Source: F1000Res. 2017 Feb 9;6:43. Originally published 2017 Jan 13. [Version 2] doi: 10.12688/f1000research.10568.2 (PMC5310529; doi:10.12688/f1000research.10568.2)
Supplement: Peer review history

# Peer review: Referee response for version 2

**DOI:** 10.5256/f1000research.11669.r19460

**Published:** 2017-03-10

**Authors:** Leonard K. Kaczmarek

**Affiliations:** 1 Department of Pharmacology, Yale School of Medicine, New Haven, CT, USA

The data presented build on the authors’ earlier studies of reversal of apoptosis. They provide useful information documenting changes in gene expression following 5 hours of exposure to ethanol, an apoptotic stimulus to primary mouse liver cells. The findings only make sense when taken in combination with the authors previous work, which provides a compelling case that the recovery from apoptosis in indeed occurring, and the manuscript documents some of this by reprinting figure panels from an earlier publication in Figure 1.  

 My only minor comment is that, at least in some cells such as neurons, cleavage of caspase-3 can be a regulatory signal that does not herald cell death, but may influence biological processes such as synaptic function.

I have read this submission. I believe that I have an appropriate level of expertise to confirm that it is of an acceptable scientific standard.

# Peer review: Referee response for version 2

**DOI:** 10.5256/f1000research.11669.r20082

**Published:** 2017-02-10

**Authors:** Takafumi Miyamoto

**Affiliations:** 1 University of Tokyo, Tokyo, Japan

The authors sincerely responded to my comment with deep consideration. Therefore I have no objection to this article being indexed.

I have read this submission. I believe that I have an appropriate level of expertise to confirm that it is of an acceptable scientific standard.

# Peer review: Referee response for version 1

**DOI:** 10.5256/f1000research.11388.r19503

**Published:** 2017-01-30

**Authors:** Sanzhen Liu

**Affiliations:** 1 Department of Plant Pathology, Kansas State University, Manhattan, KS, USA

The manuscript by Tang et al. was focused on the elucidation of the molecular mechanisms of an important phenomenon, anastasis, through time-course expression profiling. Anastasis was recently discovered and has not been fully studied yet. It's molecular basis remains to be uncovered. The study provided useful information to better understand this underexplored process. Overall, the experiment was well designed. The time course experiment included six time points, untreated samples as the control, toxin-induced apoptosis, and four time points after removal of toxin. Three biological replicates were performed at each time point. Figure 1 illustrated the experimental design very well. The biological interpretation of microarray results is reasonable. The reviewer has no major concerns. However, several minor changes are needed, especially for the presentation of figures, which could be improved.

 First, no multiple test correction was mentioned in the microarray analysis section. It was described that the p-value less than 0.05 was used to declare statistical significance. The reviewer would suggest the authors confirm that. A false discovery rate (FDR) method is needed for multiple test correction.

 Second, the PCA result from Figure 2A showed that three biological replicates were closely clustered, which showed a good repeatability. However, the goal of PCA is not just check the repeatability of three replicates of each group (time point). PCA can be also used to examine the relationship among groups. My recommendation is that the authors provide more description for the PCA result. In addition, in Figure 2A, the percentage of PC2 explaining total variation was masked. But based on the value of PC3, it should be greater than 5.89%. Given the high value of PC1, I would suggest plotting a two dimension PCA plot to display the result or re-plotting this three-dimension plot.

 Third, it would be useful to list the number of significant differential expression for each comparison. And I guess the clustering result in Figure 3 presented all significant genes.

 Figure 4 showed some interesting result about gene interactions. I did not see enough description about this figure in the main text.

 Editorial comments:

 In the Abstract, “whole genome” can be replaced by “genome-wide”.

I have read this submission. I believe that I have an appropriate level of expertise to confirm that it is of an acceptable scientific standard.

# Peer review: Referee response for version 1

**DOI:** 10.5256/f1000research.11388.r19354

**Published:** 2017-01-20

**Authors:** Takafumi Miyamoto

**Affiliations:** 1 University of Tokyo, Tokyo, Japan

This study unravels the gene regulatory network that seems to be involved in the process of anastasis. It is interesting that the authors found various genes that appear to participate in ethanol-induced anastasis, suggesting that the dynamic reconstitution of gene regulatory networks might be a prerequisite for rescuing cells from the brink of cell death. Overall, this work is worth being indexed. However, I would like to see the following points in the research addressed, before approval:

1. Anastasis is a developing concept rather than an established one. It would be better to show the expression dynamics of caspase-3, PARP, and ICAD at all analyzed time points (Cont, R0, R3, R6, R24, and R48). In addition, why don't the authors show apoptotic DNA fragmentation to make sure that all the analyzed cells in the anastasis stage definitely underwent apoptosis?
2. I may have missed noting this, but there is no statistical analysis of the gene expression changes observed in the microarray data. In Fig. 2B, the expression levels of several genes seem different in the same time point replicates. It would be better to show the genes that were induced or suppressed during anastasis, along with the statistical significance of the differences.
3. Given the importance of understanding the mechanism of anastasis, it would be better to verify the data obtained from microarray analysis, by using quantitative PCR or Western blotting.

I have read this submission. I believe that I have an appropriate level of expertise to confirm that it is of an acceptable scientific standard, however I have significant reservations, as outlined above.

# Response

**Published:** 2017-02-03

**Authors:** Ho Lam Tang

**Affiliations:** Johns Hopkins University, USA

We thank for the enthusiasm and valuable input from the reviewer, and have made the following changes:

1. We have included the Western blot data (Figure 1B), which shows that caspase-3, PARP and ICAD were cleaved during apoptosis, but then recovered to their original level at 24 hours after removal of the cell death stimulus. Interestingly, our microarray data shows that their level of mRNA remained no significant change at all time points (3, 6, 24 and 48 hours) after removal of the cell death stimulus, compared with the untreated (control) cells (data available at figshare, please see *Data availability* in the manuscript), suggesting the recovery of corresponding proteins is contributed by the regulation of translation during and after anastasis. The related data and discussion are included in our revised manuscript.
  Our earlier studies using time-lapse live cell microscopy and comic assay demonstrated that the current apoptotic induction (4.5% ethanol, 5 hours) can trigger DNA damage. After removal of the stimulus, major of the dying cells can recover. Interestingly, some cells that reversed apoptosis display chromosomal abnormality and oncogenic transformation, indicating reversibility of apoptosis after DNA damage. In our current study, we further found significant reduction of mRNA level of multiple histone genes during anastasis. Notably, cellular levels of histones reduce in response to DNA damage, as to enhance DNA repairing. Therefore, reduction of expression of histones during anastasis could be a sign of cells that recover from DNA damage after apoptosis. 
2. We have included supplementary data with corresponding p-value for statistical significance of fold change for all of the 3 biological replicants of each gene (see Data availability). The software for the microarray data analysis is mentioned at the “Materials and methods” section.
3. We have verified our data by RT-PCR in human liver cancer HepG2 cell line, and included the data in the new Figure 4.
